# The recombinant expression systems for structure determination of eukaryotic membrane proteins

**DOI:** 10.1007/s13238-014-0086-4

**Published:** 2014-08-15

**Authors:** Yuan He, Kan Wang, Nieng Yan

**Affiliations:** 1 State Key Laboratory of Bio-membrane and Membrane Biotechnology, Tsinghua university, Beijing, 100084 China; 2 Center for Structural Biology, School of Medicine, Tsinghua university, Beijing, 100084 China; 3 China-Japan Friendship Hospital, Beijing, 100029 China

**Keywords:** eukaryotic membrane proteins, recombinant expression, structural biology, integral membrane proteins (IMPs), fluorescence detected size exclusion chromatography (FSEC), protein purification and crystallization

## Abstract

Eukaryotic membrane proteins, many of which are key players in various biological processes, constitute more than half of the drug targets and represent important candidates for structural studies. In contrast to their physiological significance, only very limited number of eukaryotic membrane protein structures have been obtained due to the technical challenges in the generation of recombinant proteins. In this review, we examine the major recombinant expression systems for eukaryotic membrane proteins and compare their relative advantages and disadvantages. We also attempted to summarize the recent technical strategies in the advancement of eukaryotic membrane protein purification and crystallization.

## Introduction

It is estimated that approximately 30% of the protein-coding genes are for integral membrane proteins (IMPs) in human (Overington et al., 2006; Murray et al., 2012). IMPs are critical players for many important physiological processes including metabolism, signal transduction, and energy conversion and utilization (Krogh et al., 2001). Aberrant expressions and activities of IMPs are associated with a variety of diseases such as cancer, Alzheimer’s disease, and metabolic diseases (Ishikawa et al., 2004; Sanders and Myers, 2004; Overington et al., 2006; Aisenbrey et al., 2008; Bkaily and Al-Khoury, 2014). IMPs constitute more than 50% of the US Food and Drug Administration (FDA)-approved drug targets (Russell and Eggleston, 2000; Yildirim et al., 2007). Structures of eukaryotic membrane proteins are actively pursued for structure-based drug development.

**Figure 1:**
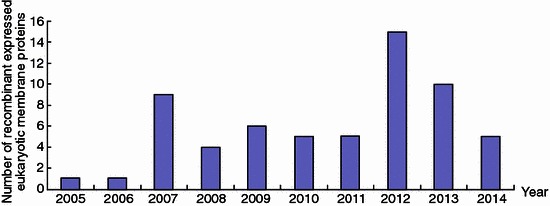
**The development trends in recombinant expression eukaryotic membrane proteins**. The structure number of eukaryotic membrane protein is limited by some obstacles such as low yield and instability in detergents. Since the first eukaryotic membrane protein structure was determined in 2005, over sixty structures have been emerged until now

In contrast to their physiological and pathophysiological significance, the progress on the structure biology of IMPs, particularly eukaryotic IMPs, has been relatively slow. By the end of March 2014, in total 466 unique membrane protein structures have been reported (Snider and Stephen, 2014), the majority of which are of prokaryotic origins. With respect to eukaryotic IMPs, more than half of the determined structures are for proteins obtained from endogenous sources (Bill et al., 2011). These proteins, exemplified by the electron transport chain complexes (Tsukihara et al., 1996; Xia et al., 1997; Sun et al., 2005), ATP synthases (Abrahams et al., 1994; Liu et al., 2004; Amunts et al., 2007), and photosystems (Kurisu et al., 2003; Liu et al., 2004; Amunts et al., 2007), usually exist in abundance and are biochemically stable, hence representing ideal candidates for structural analysis. However, the total types of endogenously abundant eukaryotic IMPs are limited. The majority of IMPs exist in low copies in the host species. Therefore, structural determination of most eukaryotic IMPs requires recombinant expression of the target proteins. The first atomic-resolution structure of a eukaryotic IMP obtained through recombinant expression, Kv1.2, was reported in 2005 (Long et al., 2005). Ever since, less than seventy structures have been obtained for eukaryotic IMPs generated through recombinant expression systems (Fig. 1).

Out of the many challenges facing structural study of eukaryotic IMPs, production of sufficient quantities of well-behaved recombinant proteins represents the real technical bottleneck. Embedded in lipid bilayers, the structural integrity and proper functions of IMPs rely on the interactions with surrounding lipids (Phillips et al., 2009), which stabilize membrane proteins, provide lattice contacts, and in some occasions function as indispensable co-factors (van Meer et al., 2008). Recombinant expression of membrane proteins therefore requires a proper membrane environment. Whereas *Escherichia coli* proved to be the best host for most of prokaryotic IMPs of known structures, eukaryotic IMPs, with very few exceptions, requires eukaryotic expression systems including yeast, baculovirus-infected insect cells, and mammalian cells (Bill et al., 2011; Snider and Stephen, 2014).

In this review, in the hope of extracting some general principles on the expression and crystallization of eukaryotic membrane proteins, we examine the expression systems for the eukaryotic IMPs whose structures are obtained, attempt to summarize and compare the advantages and disadvantages of the representative recombinant expression systems, and delineate the detailed information in eukaryotic membrane protein purification and crystallization (Table 1).

**Table 1:** Expression, purification, and crystallization information for eukaryotic membrane proteins*

No.	Expression systems		Protein name	Family	Extraction detergent	Purification detergent	Gel filtration detergent	Final concentration (mg/mL)	Temperature (°C)	Methods
1	*E. coli*	Bl21(DE3)	FLAP	MAPEG	DDM	DDM	C_12_E_8_ + C_8_E_4_			Sitting drop
2		C43(DE3)	PfAQP	Water channel	OG	OG	OG	6	18	Hanging drop
3		Bl21(DE3)	Kir3.1-prokaryotic Kir channel chimera	Potassium channel	DDM	DDM	NG	8	20	Sitting drop
4		Bl21(DE3)	Cytochrome b_561_	Electron transport chain complexes	DM	NM	NG		18	Hanging drop
5	Yeast	*Pichia Pastoris*	Kv1.2 with β subunit	Potassium channel	DDM	DDM	DM	10	20	Hanging drop
6			Kv1.2-Kv2.1paddle	Potassium channel	DDM	DDM	CYMAL6 + CYMAL7	10	20	Hanging drop
7			Kv2.1paddle-Kv1.2 (F233 W)	Potassium channel	DDM	DDM	DDM	10	20	Hanging drop
8			Kir2.2 inward-rectifier	Potassium channel	DM	DM	DM	8	20	Hanging drop
9			GIRK2 (Kir3.2) K^+^ channel	Potassium channel	DM	DM	DM	6–7	20	Hanging drop
10			K_2P_1.1(KWIK-1)	Potassium channel	DDM	DDM	DDM	10	20	Hanging drop
11			K_2P_4.1(TRAAK)	Potassium channel	DDM	DDM	DM	5	4	Hanging drop
12		*Pichia Pastoris*	Calcium release-activated calcium channel	Calcium channel	DDM	DDM	NM + NG	16	17	Hanging drop
13			SoPIP2;1	Water channel	OG	OG	OG	10	4	Hanging drop
14			HsAQP5	Water channel	NG	NG	NG	10	8	Hanging drop
15	Yeast		HsAQP4	Water channel	OG	OG	OG	30	25	Hanging drop
16			P-Glycoprotein	ABC transporter	Triton	DDM	DDM	10	4	Sitting drop
17			P-Glycoprotein	ABC transporter	DDM	DDM	UDM		4	Hanging drop
18			LTC_4_S	MAPEG	DDM	DDM	DDM	6.5	4	Sitting drop
19			Histamine H_1_ receptor	GPCR	DDM	DDM	DDM	30–40	20	LCP
20		*S. cerevisiae*	AHA2 (H^+^ pump)	Pump	DDM	DDM	C12E8 + CYMAL5	20–30	4	Hanging drop
21			VrH^+^-Ppase	M-PPase	DDM	DM	DM	10	20	Hanging drop
22			NRT1.1	MFS transporter	DDM	DDM	DDM	10	4	Hanging drop
23			CAAX protease Ste24p	Intramembrane protease	DDM	DDM/C_12_E_7_	C_12_E_7_	7.35	4/17	Hanging drop
24			PiPT	MFS transporter	DDM	DDM	NG	10–15	20	Hanging drop
25	Insect cell	*S. frugiperda*	β_2_AR (Fab)	GPCR	DDM	DDM	DDM	8–12	22	Bicelle
26			β_2_AR (T4L)	GPCR	DDM	DDM	DDM	Concentrated	22	LCP
27			β_2_AR-agonist complex	GPCR	MNG	MNG	MNG (0.1%)	50	20	LCP
28			β_2_AR-GS complex	GPCR	MNG	MNG	MNG (0.1%)	90	20	LCP
29			A2A adenosine receptor	GPCR	DDM	DDM	DDM	70	20	LCP
30			CXCR4	GPCR	DDM	DDM	DDM	60–70	20	LCP
31			Dopamine D3 receptor	GPCR	DDM	DDM	DDM	20–30	20	LCP
32			Sphingosine 1-phosphate receptor	GPCR	DDM	DDM	DDM	100	20	LCP
33			M2 muscarinic acetylcholine receptor	GPCR	Digitonin + Na-cholate	DM	MNG	30	20	LCP
34			M3 muscarinic acetylcholine receptor	GPCR	DDM	DDM	MNG	60	20	LCP
35			κ-Opioid receptor	GPCR	DDM	DDM	DDM	40	20	LCP
36			μ-Opioid receptor	GPCR	DDM + CHAPS + CHS	DDM + CHAPS + CHS	MNG + CHS	30	20	LCP
37			δ-Opioid receptor	GPCR	MNG + CHAPS + CHS	MNG + CHAPS + CHS	MNG + CHAPS + CHS	50	20	LCP
38	Insect cell	*S. frugiperda*	N/OFQ receptor	GPCR	DDM + CHS	DDM + CHS	DDM + CHS	40	20	LCP
39			CCR5	GPCR	DDM + CHS	DDM + CHS	DDM + CHS	40–50	20	LCP
40			PAR1	GPCR	DDM + CHS	DDM + CHS	DDM + CHS	40–50	20	LCP
41			5-HT_1B/2B _serotonin receptor	GPCR	DDM + CHS	DDM + CHS	DDM + CHS	50–80	20	LCP
42			Smoothened receptor	GPCR	DDM + CHS	DDM + CHS	DDM + CHS	50–60	20	LCP
43			Glucagon receptor	GPCR	DDM + CHS	DDM + CHS	DDM + CHS	80	20	LCP
44			Metabotropic Glutamate Receptor 1	GPCR	DDM + CHS	DDM + CHS	DDM + CHS	50–80	20	LCP
45			P2X4	Channel	DDM	DDM	DDM	2	4	Hanging drop
46			ASIC1	Channel	DDM	DDM	DDM	5	4	Hanging drop
47			GluA2	Channel	DDM	DDM	C_11_Thio + lipids	2	4	Hanging drop
48			GLuCla	Cys-loop receptor	DDM	DDM	DDM	2	4	Hanging drop
49			CX26	Gap junction	DDM	DDM	UDM	30	4	Hanging drop
50			UT-B	Urea Transporter	DM	DM	OG	8	4	Sitting drop
51			ZMPSTE24	Intramembrane protease	DDM + CHS or OGNG + CHS	DDM + CHS or OGNG + CHS	DDM + CHS or OGNG + CHS	9–11	20	Sitting drop
52	Insect cell		ABCB10	ABC transporter	DDM	DDM	DDM		20	Sitting drop
53			Claudin-15	Tight junction	DDM	LMNG	LMNG	7	20	LCP
54			NRT1.1	MFS transporter	DDM	DDM	DDM	10	4	Hanging drop
55		*Trichoplusiani*	β1 adrenergic receptor	GPCR	DM	Octylthioglucoside	Octylthioglucoside	6	18	Hanging drop
56			NTS1 Neurotensin Receptor	GPCR	MNG + CHS	MNG + CHS	MNG + CHS	20–25	20	LCP
57			CmClC	H^+^/Cl^-^ exchange transporter	DM	DM	DM	10	20	Hanging drop
58			Corticotropin-releasing factor receptor	GPCR	DM	DM	DM	20–30	22.5	LCP
59			GLUT1	MFS transporter	DDM	DDM	NG	10	4	Hanging drop
60		COS-1 cells	Rhodopsin	Rhodopsin	DDM	DDM	C_8_E_4_			
61	Mammalian cell	HEK293	RhCG	Channel	OG	OG	OG	5	20	Hanging drop
62			Dopamine transporter	Solute carrier transpoter	DDM	DDM CHS POPC:POPE:POPG = 3:1:1	DM CHS POPE	3	4	Hanging drop

* The blank in the table is due to the details in the reported method has not been mentioned

## Recombinant expression systems for eukaryotic membrane proteins

The recombinantly expressed eukaryotic IMPs of known structures were obtained from four systems: *E. coli*, yeasts (*Pichia Pastoris* and *Saccharomyces cerevisiae*), insect cells, and mammalian cells. These expression systems have their respective advantages and disadvantages. The choice of an appropriate expression system remains empirical, largely depending on the biochemical and biological properties of the target proteins (Bernaudat et al., 2011). Among the recombinantly expressed eukaryotic IMPs whose structures have been solved, 4 were expressed in *E. coli*, 20 in yeast, 35 in insect cells, and 3 in mammalian cells. Below we will discuss these four expression systems.

### *E. coli*

As the most frequently exploited recombinant expression system, *E. coli* BL21 (DE3) has the obvious advantage of rapid replication, time-saving operation, inexpensive cost, and mature and easy genetic manipulations (Sahdev et al., 2008). *E. coli* C43 (DE3) and C41 (DE3) strains were developed for over-expression of membrane proteins (Miroux and Walker, 1996; Dumon-Seignovert et al., 2004). Indeed, these *E. coli* strains were employed to over-express the large majority of prokaryotic IMPs whose structures were finally obtained. However, as the prokaryotic expression systems, they may lack the essential lipids, molecular chaperons, and post-translational modifications that are required for the correct membrane insertion, folding, and function of eukaryotic IMPs (Sahdev et al., 2008). As a result, only 4 structures were obtained for eukaryotic IMPs expressed in *E. coli* (Table 2). Despite the challenge to express eukaryotic membrane proteins in *E. coli*, researchers attempted to overcome these hurdles with codon-optimization (Burgess-Brown et al., 2008) and protein fusion with Mistic or GlpF tag to promote protein expression (AegeanSoftware, 2005; Drew et al., 2006; Neophytou et al., 2007), and co-expression of post-translational machineries to facilitate protein folding (Mironova et al., 2005; Mijakovic et al., 2006). Regardless of the effort, *E. coli* may not be an ideal system for eukaryotic IMP expression.

**Table 2:** *E. coli* as an expression system for eukaryotic membrane protein

Expression systems		No.	Protein	Species	PDB code	Reference
*E. coli*	Bl21(DE3)	1	FLAP	*Homo sapiens*	2Q7 M 2Q7R	Ferguson et al., 2007
	C43 (DE3)	2	PfAQP	*Plasmodium falciparum*	3C02	Newby et al., 2008
	Bl21(DE3)	3	Kir3.1-prokaryotic Kir channel chimera	*Streptomyces lividans*	2QKS	Nishida et al., 2007
	Bl21(DE3)	4	Cytochrome b_561_	*Arabidopsis thaliana*	4O6Y, 4O79, 4O7G	Lu et al., 2014

### Yeast

Among the many yeast species, *Pichia Pastoris* (*Pichia*) and *Saccharomyces cerevisiae* (*S. cerevisiae*), which have been genetically well characterized, are the major systems to overexpress eukaryotic IMPs (Strausberg and Strausberg, 2001; Bornert et al., 2012). *Schizosaccharomyces pombe* is also employed for overexpression of IMPs, but not as widely as *Pichia* and *S. cerevisiae* (Yang et al., 2009). During the past thirty years, yeast has proved to be a useful expression system: 15 eukaryotic IMP structures have been determined for proteins expressed in *Pichia* expression system and 5 by *S. cerevisiae*. Most of the structurally available eukaryotic channels such as potassium channels and water channels were expressed in yeast, as listed in Table 3.

**Table 3:** Yeast as an expression system for eukaryotic membrane protein*

Expression systems		No.	Protein	Species	PDB code	Reference
Yeast	*Pichia Pastoris*	1	Kv1.2 with β subunit	*Drosophila melanogaster*	2A79	Long et al., 2005
		2	Kv1.2-Kv2.1 paddle	*Rattus norvegicus*	2R9R	Long et al., 2007
		3	Kv2.1paddle-Kv1.2 (F233 W)	*Rattus norvegicus*	3LNM	Tao et al., 2010
		4	Kir2.2 Inward-Rectifier	*Gallus gallus*	3JYC	Tao et al., 2009
		5	GIRK2 (Kir3.2) channel	*Mus musculus*	3SYO	Whorton and MacKinnon, 2011
		6	K_2P_1.1 (KWIK-1)	*Homo sapiens*	3UKM	Miller and Long, 2012
		7	K_2P_4.1 (TRAAK)	*Homo sapiens*	3UM7	Brohawn et al., 2012
		8	Calcium release-activated calcium channel	*Drosophila melanogaster*	4HKR	Xiaowei Hou, 2012
		9	SoPIP2;1	*Spinacia oleracea*	1Z98 2B5F	Tornroth-Horsefield et al., 2006
		10	HsAQP5	*Homo sapiens*	3D9S	Horsefield et al., 2008
		11	HsAQP4	*Homo sapiens*	3GD8	Ho et al., 2009
		12	P-Glycoprotein	*M. musculus*	3G5U, 3G60, 3G61	Aller et al., 2009
		13	P-Glycoprotein	*Caenorhabditis elegans*	4F4C	Jin et al., 2012
		14	LTC4S	*Homo sapiens*	2PNO	Ago et al., 2007
		15	Histamine H_1_ receptor	*Homo sapiens*	3RZE	Shimamura et al., 2011
	*S. cerevisiae*	16	AHA2 (H+ pump)	*Arabidopsis thaliana*	3B8C	Pedersen et al., 2007
		17	VrH+-Ppase	*Vigna radiata*	4A01	Lin et al., 2012
		18	NRT1.1	*Arabidopsis thaliana*	4CL4	Parker and Newstead, 2014
		19	CAAX protease Ste24p	*Saccharomyces mikatae*	4IL3	Pryor et al., 2013
		20	PiPT	*Piriformospora indica*	4J05	Pedersen et al., 2013

* For some proteins like GPCR and potassium channel, only the representative ones are listed

*Pichia* is considered the best expression system among yeast species (Cereghino and Cregg, 2000). Several elements contribute to its increasing applications, including the simplicity of genetic manipulation, the high yield of heterologous protein, the cost-effective chemical reagents, as well as the ability of post-translational modifications (Macauley-Patrick et al., 2005). For these reasons, *Pichia* is a more suitable expression system for producing eukaryotic IMP than *E. coli*. *Pichia* shares the advantage of the molecular and genetic manipulation with *S. cerevisiae*, yet it adds extra advantage of 10- to 100- fold biomass out of the same cultural volume comparing with *S. cerevisiae* (Macauley-Patrick et al., 2005).

The improved techniques and the commercial availability together promote the development of *Pichia* (Cereghino and Cregg, 2000). *Pichia* is a methylotrophic yeast, capable of utilizing methanol as its sole carbon source (Yurimoto and Sakai, 2009). A promoter derived from the alcohol oxidase I (*AOXI*), which is the first-step enzyme in the methanol metabolism, strictly controls the expression of the foreign proteins (Macauley-Patrick et al., 2005). The commercial vector pPICZ (or pPICZα) takes advantage of the *AOXI* promoter, being induced by methanol (Li et al., 2007). *AOXI* promoter is prevailing than other promoters like *PMA1* and *GPD1* for its strong and highly inducible ability (Cereghino and Cregg, 2000). After the vector is readily prepared and transformed into the competent cells, the target gene can be inserted into the *Pichia* genome in high efficiency via homologous recombination to generate stable cell lines, and then the colonies with multiple copies that exhibit the highest protein expression level will be screened out through zeocin-spread plates (Daly and Hearn, 2005). This zeocin selective marker for transformation selection is important regarding to the convenience of genetic manipulation in yeast. All the procedure typically takes about 10–15 days for a complete procedure from subcloning to protein expression. A potential disadvantage of the yeast culture concerns the difficulty in cell disruption due to the thick and hard cell walls.

### Insect cell

The baculovirus infected insect cell system is undoubtedly the dominant heterologous expression system for obtaining eukaryotic IMPs (Contreras-Gomez et al., 2014). The most common method for generating recombinant baculovirus is based on the site-specific transposition of an expression cassette into a baculovirus shuttle vector (bacmid) that is amplified in *E. coli* (Ciccarone et al., 1998). The process is very convenient: clone the target gene into the pFastBac vector which uses the strong AcMNPV polyhedron (PH) as the promoter for high level protein expression, then transform the pFastBac vector into DH10Bac *E. coli* competent cells. DH10Bac cells possess a baculovirus shuttle vector (bacmid) with a transposon site and a helper plasmid, thus can help the pFastBac vector to have a transposition on the bacmid. Once the transposition occurs and the recombinant bacmid is generated, the bacmid could be isolated and purified for transfection. After the insect cells are cultured into a desired confluence, they are transfected by the purified bacmid DNA to generate a recombinant baculovirus that used for preliminary expression test (Contreras-Gomez et al., 2014). The pFastBac is ampicillin resistance and Bacmid is kanamycin resistance, and these selective markers provide expedience for this baculovirus expression system. It takes approximately 3–4 weeks to complete these procedures for initial protein expression test.

There are two most popular insect cell lines used for IMP expression, *Spodoptera frugiperda* (Sf9) and *Trichoplusia ni* (Hi5). Heterologous proteins have disparate performances on the yield and behavior when expressed in these two cell lines (Unger and Peleg, 2012). Till now, 30 structures were obtained for eukaryotic IMPs from Sf9 expression system and 5 from Hi5 (Table 4).

**Table 4:** Insect cell as an expression system for eukaryotic membrane protein*

Expression systems		No.	Protein	Species	PDB code	Reference
Insect cell	*S. frugiperda*	1	β_2_AR (Fab)	*Homo sapiens*	2R4R 2R4S	Rasmussen et al., 2007
		2	β_2_AR (T4L)	*Homo sapiens*	2RH1	Cherezov et al., 2007
		3	β_2_AR-agonist complex	*Homo sapiens*	3PDS	Rosenbaum et al., 2011
		4	β_2_AR-GS complex	*Homo sapiens*	3SN6	Rasmussen et al., 2011a,b
		5	A_2A_ adenosine receptor	*Homo sapiens*	3EML	Jaakola et al., 2008
		6	CXCR4	*Homo sapiens*	3ODU 3OE8	Wu et al., 2010
		7	Dopamine D3 receptor	*Homo sapiens*	3PBL	Chien et al., 2010
		8	Sphingosine 1-phosphate receptor subtype 1	*Homo sapiens*	3V2 W 3V3Y	Hanson et al., 2012
		9	M2 muscarinic acetylcholine receptor	*Homo sapiens*	3UON	Haga et al., 2012
		10	M3 muscarinic acetylcholine receptor	*Rattus norvegicus*	4DAJ	Kruse et al., 2012
		11	κ-Opioid receptor	*Homo sapiens*	4DJH	Wu et al., 2012
		12	μ-Opioid receptor	*Mus musculus*	4DKL	Manglik et al., 2012
		13	δ-Opioid receptor	*Mus musculus*	4EJ4	Granier et al., 2012
		14	N/OFQ receptor	*Homo sapiens*	4EA3	Thompson et al., 2012
		15	CCR5	*Homo sapiens*	4MBS	Tan et al., 2013
		16	PAR1	*Homo sapiens*	3VW7	Zhang et al., 2012
		17	5-HT_1B/2B_ serotonin receptor	*Homo sapiens*	4IAR 4IB4	Wang et al., 2013a, b; Wacker et al., 2013
		18	Smoothened receptor	*Homo sapiens*	4JKV	Wang et al., 2013a, b
		19	Glucagon receptor	*Homo sapiens*	4L6R	Siu et al., 2013
		20	Metabotropic glutamate receptor1	*Homo sapiens*	4OR2	Wu et al., 2014
		21	P2X_4_	*Danio rerio (Zebra fish)*	3I5D 3H9 V 4DW1	Kawate et al., 2009; Hattori and Gouaux, 2012
		22	ASIC1	*Gallus gallus*	2QTS 3HGC	Jasti et al., 2007; Gonzales et al., 2009
		23	GluA2	*Rat*	3KG2 3KGC	Sobolevsky et al., 2009
		24	GLuClα	*Caenorhabditis elegans*	3RHW, 3RIF, 3RI5 3RIA	Hibbs and Gouaux, 2011
		25	CX26	*Homo sapiens*	2ZW3	Maeda et al., 2009
		26	UT-B	*Bos taurus*	4EZC 4EZD	Levin et al., 2012
		27	ZMPSTE24	*Homo sapiens*	4AW6	Quigley et al., 2013
		28	ABCB10	*Homo sapiens*	4AYT	Shintre et al., 2013
		29	Caludin-15	*Mus Musculus*	4P79	Suzuki et al., 2014
		30	NRT1.1	*Arabidopsis thaliana*	4OH3	Sun et al., 2014
	*Trichoplusia ni*	31	β1 adrenergic receptor	*Meleagris gallopavo*	2VT4	Warne et al., 2008
		32	NTS1 Neurotensin Receptor	*Rattus norvegicus*	4GRV	White et al., 2012
		33	CmClC	*Cyanidioschyzonmerolae*	3ORG	Feng et al., 2010
		34	Corticotropin-releasing factor receptor	*Homo sapiens*	4K5Y	Hollenstein et al., 2013
		35	GLUT1	*Homo sapiens*	4PYP	Deng et al., 2014

* For some proteins like GPCR and potassium channel, only the representative ones are listed

After the protein IL-2 was first expressed in large scale with the baculovirus-infected insect cells in 1985, this system has been quickly accepted and widely used (Smith et al., 1983; Maeda et al., 1985). Owing to the convenience of scale up, safety and accuracy (Kost et al., 2005), the baculoviral insect cell system has yielded the largest number of eukaryotic IMPs up to date (Table 4). Notably, among the 35 eukaryotic IMP structures, 23 are of G-protein coupled receptors (GPCR) (Table 4). The insect cell system has been the prevailing expression system for eukaryotic IMP. However, the cost for the cultural medium may represent a serious roadblock for most laboratories.

### Mammalian cell

Mammalian expression system has become one of the popular recombinant protein production systems for its proper post-translational modification and human protein-like structure assembly (Khan, 2013). HEK (human embryo kidney) and CHO (Chinese hamster ovary) are two broadly used cell lines for recombinant expression. These two cell lines are extensively applied by researchers to do functional assay such as the electrophysiological assay (Kawate et al., 2009). Both these two cell lines can be applied for transient and stable transfections (Zhu, 2012). For the transient transfection approach, it is relative easier to reach to a reasonable protein expression level, but this expression level may vary from batch to batch. On the other hand, although the proteins have higher productivity and less variation in the stable transfection method, it is very time consuming (one month at least) (Condreay et al., 1999; Baldwin et al., 2003). Consequently, it is a balance for scientists to choose between these two transfection methods.

HEK293 is a specific cell line originally derived from HEK cells, while the number “293” comes from Graham’s habit of numbering his experiments (Louis et al., 1997). Large scale, transient transfection of HEK293 in suspension culture is a reliable way to generate milligram quantities of recombinant eukaryotic IMPs. When the gene of interest is ligated into the vector pcDNA3 or pCMV5, the complete plasmid is then transfected into the HEK293 cells and the cells are harvested after 48 h (Thomas and Smart, 2005). The whole procedure is more or less similar to that of the insect cell system, only with a couple of exceptions. For example, 5%–10% CO_2_ is required for maintaining the HEK293 cells, and the culture temperature is 37°C for HEK293 but not 27°C as for insect cells. The overall process usually requires one to two weeks from initial cloning to small scale test for the transient expression. However, ascribe to the low yield, slow growth rate and higher cost of complex media (Sunley and Butler, 2010), the number of eukaryotic IMP structures generated based on the mammalian cells is very limited. So far, only three eukaryotic IMP structures are from this system, and two of them are obtained from HEK293 cells (Table 5).

**Table 5:** Mammalian cell as an expression system for eukaryotic membrane protein

Expression system		No.	Protein	Species	PDB code	Reference
Mammalian	HEK293	1	Rodopsin	*Homo sapiens*	2J4Y	Standfuss et al., 2007
		2	RhCG	*Homo sapiens*	3HD6	Gruswitz et al., 2010
		3	Dopamine transporter	*Homo sapiens*	4M48	Penmatsa et al., 2013

The BacMam system has to be mentioned for its safety, reproducibility and efficiency (Dukkipati et al., 2008). The baculoviruses are engineered by inserting a mammalian expression cassette for delivering foreign genes in mammalian cells. Their non-replicating property makes they are safe and well-tolerated by mammalian cells. BacMam system gains widespread use for its safety and rapid manipulation (Reeves et al., 2002; Baconguis and Gouaux, 2012). Depending on the cell type, cell division rate and transduction efficiency, it lasts 5–14 days to detect the gene expression (Dukkipati et al., 2008). The dopamine transporter structure was determined by the BacMam system (Penmatsa et al., 2013).

From the foregoing discussion, it is concluded that every expression system has their distinctive properties for protein expression. We compare their relative merits for an intuitional understanding of each system which can help researchers to make the best choice for their proteins expression (Table 6).

**Table 6:** Comparison among four expression system

	*E. coli*	Yeast (*Pichia*)	Insect cell (Sf9)	Mammalian cell (HEK293)
Duration time before cell cultivation (Days)	3–5	6–8	25–30	Transient: 3–5 Stable: at least 30
Cell cultivation time for 1L test (Days)	1–2	3–7	2–4	2–4
Cost for 1L test ($) in China	15–20	20–25	200–250	200–250
Number of available eukaryotic IMP structures	4	20	34	3

## Homologue screen

Eukaryotic membrane proteins are very difficult to yield in large quantities, and most of them tend to be unstable in the presence of detergents. As a result, identification of well-expressed proteins is very essential. Homologue screen is widely applied for researchers to discover well-behaved proteins (Kawate et al., 2009; Xiaowei Hou, 2012).

Fluorescence detected size exclusion chromatography (FSEC) is a powerful method for homologue screen (Drew et al., 2006; Newstead et al., 2007). Compared with the common protocols, GFP fusion membrane proteins can be detected by measuring fluorescence in whole cells during the over-expression process. It saves time to help people preclude proteins that have no expression or low expression level. Also, it is much easier to assess the integrity of proteins by detecting the fluorescence in SDS polyacrylamide gels. Moreover, FSEC could be employed to figure out the most stable detergents in initial detergent screen. Considering these benefits, this technology is very widely applied (Jasti et al., 2007; Gonzales et al., 2009; Kawate et al., 2009; Sobolevsky et al., 2009). Taking P2X receptor as an example (Kawate et al., 2009), because of its aggregation and instability problems, researchers applied this method to screen 35 orthologs and finally got one species which was fit for crystallization. FSEC is proven to be one of the most robust methods to facilitate the identification of appropriate candidates for solving the structures of eukaryotic membrane proteins.

## Optimal constructs design

Optimizing constructs is very beneficial for getting the well-packed crystals. One way for optimizing constructs is to “cut off”. Limited proteolysis is a conventional method to find the optimal constructs. Besides, it is worth noting that either N-terminal tag or C-terminal tag is removed before crystallization in most crystallization cases (Long et al., 2005; Long et al., 2007; Gonzales et al., 2009; Maeda et al., 2009; Sobolevsky et al., 2009; Tao et al., 2009). For instance, the desensitized ASIC1 was crystallized by removal of 25 N-terminal and 64 C-terminal residues (Jasti et al., 2007).

The contrary way for optimizing constructs is to “add up”. T4 lysozyme (T4L) insertion and Fab/nanobody replacement are applied to produce stable proteins. The T4L fragment is soluble enough to effectively increase the solvent-exposed area, thereby facilitating protein-protein interactions and generating novel crystal packing interfaces (Cherezov et al., 2007). Fab/nanobody, which are generated from monoclonal antibodies, can reduce the protein flexibility and improve the conformational homogeneity (Zhou et al., 2001; Rasmussen et al., 2007). GPCR is one of the most successful cases employing T4L and Fab/nanobody to the ultimate structure determination (Rasmussen et al., 2007; Rasmussen et al., 2011a, b).

Mutagenesis is an alternative way for constructs design. In order to improve the crystallization behavior and stabilize the tetrameric state of the glutamate receptor GluA2, point mutations were introduced, preventing non-specific aggregation and disulphide bond formation (Sobolevsky et al., 2009). And E329Q was introduced in order to stabilize GLUT1 in a certain conformation (Deng et al., 2014). Plus, glycosylation is the most common post-translational modification of eukaryotic membrane proteins and leads to heterogeneity of proteins. Thus, mutating of glycosylation sites or deglycosylation by enzymes is an essential step for crystallization (Deng et al., 2014).

## Detergents, lipids and crystallization

**Figure 2:**
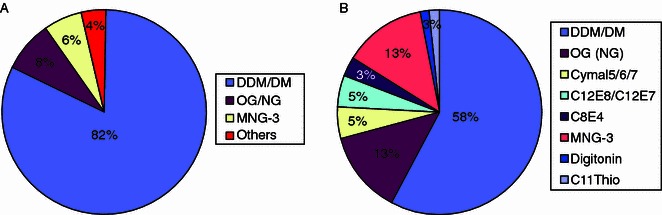
**Detergents used for extraction and crystallization of eukaryotic membrane proteins**. (A) Detergents for protein extraction and purification. DDM/DM can be applied for most eukaryotic membrane proteins in extraction step. (B) Detergents for protein crystallization. DDM/DM is the major detergent for the crystallization of eukaryotic membrane proteins

We have summarized the detergents used for protein purification and crystallization from Table 1. 51 eukaryotic membrane proteins can be extracted from DDM or DM (Fig. 2A), suggesting DDM/DM are the detergents suitable for the extraction process of the majority of eukaryotic membrane proteins. Collaterally, nearly half of the eukaryotic membrane protein crystals are obtained from DDM/DM, indicating DDM/DM are worthy of a trial for crystallization in the first place (Fig. 2B and Table 1). Apart from these conventionally applied detergents, new detergents have also been developed to meet the new requirements. For example, when purifying β_2_ adrenergic receptor-Gs protein, the authors stabilized protein complex by exchanging DDM with a newly developed maltose neopentyl glycol detergent MNG-3 (NG310, Anatrace) to prevent the complex dissociated from original detergent DDM (Chae et al., 2010; Rasmussen et al., 2011a, b).

It is worth noting that additional lipids are able to help crystal packing. There are three ways of lipid combinations. The first is mixing lipids with detergent(s) in hanging or sitting drop during crystallization. Take mammalian voltage-dependent *shaker* family potassium channel as an example, the author utilized 0.1 mg/mL 3:1:1 POPC: POPE: POPG throughout purification and crystallization to obtain crystals (Long et al., 2005). The second approach is lipid cubic phase (LCP) method. The lipid cubic phase is a dynamic structure consisting of a highly organized single lipid bilayer pervaded by an inter-connected aqueous channel (Landau and Rosenbusch, 1996). Martin has an elaborate discussion about LCP method which we will not go into details in this review (Caffrey and Cherezov, 2009). The crystal structure of β_2_AR-GS complex was determined by the use of 7.7 MAG as the host lipid for crystallization (Rasmussen et al., 2011a, b). The third way is bicelle method, which is regarded as an intermediate approach between the traditional detergent crystallization method and the rigid LCP method. Bicelle can be considered as a lipid bilayer disc that formed by a long chain lipid and a short chain lipid or detergent (Agah and Faham, 2012). The general composition is 3:1 DMPC: CHAPSO. Several protein structures were determined utilizing bicelle method (Rasmussen et al., 2007; Payandeh et al., 2011).

Last but not the least, we will elaborate a few messages for the crystallization of eukaryotic membrane protein drawn from Table 1: (a) Protein concentration: almost all the protein concentration for crystallization is above 5 mg/mL. (b) Crystallization temperature: if we expel the LCP method that is routinely crystallized at 20 ± 2°C, nearly half of the eukaryotic membrane proteins are crystallized at low temperature, especially on 4°C. At cold temperature, for protein with “normal” solubility, protein will be more soluble in high salt and precipitate from lower concentration of the precipitant reagents, and also the equilibrium diffusion rate occurs more slowly. These manifest that crystallization at lower temperature is absolutely an indispensable trial. (c) Crystallization methods: hanging drop or sitting drop crystallization method is the main and conventional approach for most eukaryotic membrane protein. LCP method is an up-rising star which is extensively applied in determining the GPCR’s structures which we have mentioned before. Remarkably, LCP method is not only propitious to GPCR, but also is able to be applied for none-GPCR protein structures determination (Suzuki et al., 2014).

## Conclusion

In this review, we discuss the benefits and drawbacks of different expression systems for eukaryotic membrane protein, and illustrate some general methods of recent advances for eukaryotic membrane protein purification and crystallization. We hope our work can provide help to those who are interested and work on eukaryotic membrane proteins. Although the discussion of eukaryotic membrane protein structure determined by Cryo-EM or NMR is beyond the scope of this review, the general methodologies and technical strategies summarized here also come to an aid in protein yield augment and sample homogeneity improvement for Cryo-EM and NMR. They are very powerful tools to solve structures, for instance, the Cryo-EM was applied to determine TrpV1 structures (Cao et al., 2013; Liao et al., 2013). With the development of advanced technologies, more and more eukaryotic membrane protein structures will emerge to answer the most significant questions in life sciences and provide the novel pharmaceutical targets in drug design.

## Abbreviations

β_2_AR: human β2 adrenergic G-protein-coupled receptor
ABCB10: ATP-binding cassette (ABC) transporters
AHA2: *Arabidopsis thaliana* auto-inhibited H1-ATPase 2
ASIC1: acid-sensing ion channel 1
CAAX protease Ste24p: C is cysteine redidue, A is an aliphatic residue and X is any residue. It is a zinc metalloprotease catalyzing two proteolytic steps in the maturation of yeast mating pheromone a-factor
C_8_E_4_: tetraethyleneglycol monooctyl ether
C_12_E_7_: dodecylheptaglycol
C_12_E_8_: polyoxyethylene dodecyl ether
CHS: cholesteryl hemisuccinate
CmClC: cyanidioschyzon merolae chloride (Cl^–^) ions transporter
C_11_Thio: n-undecyl-β-D-thiomaltopyranoside
CXCR4: human chemokine receptors
CX26: connexin 26 gap junction
CYMAL5: 5-cyclohexyl-1-pentyl-β-D-maltoside
CYMAL6: 6-cyclohexyl-1-hexyl-β-D-maltoside
CYMAL7: 7-cyclohexyl-1-heptyl-β-D-maltoside
DDM: n-dodecyl-β-D-maltoside
DM: n-decyl-β-D-maltoside
FLAP: 5-lipoxygenase-activating protein
GIRK2 (Kir3.2): K^+^ channel: G protein-gated K+ channels
GluA2: a-amino-3-hydroxy-5-methyl-4-isoxazole propionic acid (AMPA)-sensitive ionotropic glutamate receptor
GLuClα: caenorhabditis elegans glutamate-gated chloride channel a (GluCl), an inhibitory anion-selective Cys-loop receptor
HsAQP4: human aquaporin 4
HsAQP5: human aquaporin 5
Human BK channel: high-conductance voltage- and Ca21-activated K1 channels
K2P1: two-pore domain potassium (K+) channels
Kv1.2: voltage-dependent shaker family potassium channel
Kv1.2-Kv2.1 paddle: ‘paddle-chimaera channel’, voltage-sensor paddle has been transferred from Kv2.1 to Kv1.2
LTC4S: cysteinyl leukotrienes
M-Ppase: membrane-integral pyrophosphatases
MAPEG: membrane-associated proteins in eicosanoid and glutathione metabolism
MNG: maltose-neopentyl glycol
NG: n-nonyl-β-D-glucopyranoside
NM: n-nonyl-β-D-maltoside
N/OFQ receptor: nociceptin/orphanin FQ receptor
OG: n-octyl-β-D-glucoside
OGNG: octyl glucose neopentyl glycol
P2X_4_: cation-selective ion channels gated by extracellular ATP
PAR1: protease-activated receptor 1
PfAQP: *Plasmodium falciparum* aquaglyceroporin
PiPT: a Fungal (*Piriformospora indica*) high-affinity phosphate transporter
POPC: 1-palmitoyl-2-oleoyl-sn-glycero-3-phosphocholine
POPE: 1-palmitoyl-2-oleoyl-sn-glycero-3-phosphoethanolamine
POPG: 1-palmitoyl-2-oleoyl-sn-glycero-3-phosphoglycero
RhCG: rhesus C glycoprotein
SoPIP2: 1, spinach plant plasma membrane aquaporin
TRAAK: TWIK-related arachidonic acid–stimulated K^+^ channel
UDTM: n-undecyl-β-maltoside
UT-B: urea transporters-B
VrH^+^-Ppase: vigna radiate H1-translocating pyrophosphatases
ZMPSTE24: zinc metallopeptidase STE24
